# Mesenchymal Stem Cell-Derived Exosomes in Cardioprotection: A Novel Application to Prevent Myocardial Injury

**DOI:** 10.31083/j.rcm2309310

**Published:** 2022-09-13

**Authors:** Shaokang Yang, Jialin Li, Mingbo Tang, Xinliang Gao, Wei Liu, Shixiong Wei

**Affiliations:** ^1^Department of Gastric and Colorectal Surgery, General Surgery Center, The First Hospital of Jilin University, 130021 Changchun, Jilin, China; ^2^Department of Thoracic Surgery, The First Hospital of Jilin University, 130021 Changchun, Jilin, China

**Keywords:** mesenchymal stem cell, exosomes, cardioprotection, myocardial injury

## Abstract

Perioperative myocardial injury is a common complication caused by major surgery. Many pharmacological and nonpharmacological studies have investigated perioperative cardioprotection. However, the methods are insufficient to meet the increasing clinical needs for cardioprotection. The application of Mesenchymal Stem Cell-Derived Exosomes (MSC-Exos) is a novel cell-free therapeutic strategy and has significantly benefitted patients suffering from various diseases. In this review, we comprehensively analyzed the application of MSC-Exos to prevent myocardial infarction/injury by regulating inflammatory reactions, inhibiting cardiomyocyte apoptosis and autophagy, promoting angiogenesis, and mediating cardiac remodeling. Finally, we assessed the therapeutic effects and the challenges associated with the application of MSC-Exos from a clinical perspective.

## 1. Introduction 

In an aging population, many perioperative patients suffer from cerebro-cardiovascular diseases, which result in high morbidity and mortality due to perioperative myocardial infarction (PMI) during anesthesia and surgery. PMI is a severe cardiovascular complication and contributes to non-fatal myocardial infarction, non-fatal cardiac arrest, and perioperative cardiac death in around 500,000~900,000 individuals, and also increases the risk of death due to cardiovascular complications every year in the first six months after major non-cardiac surgery [1, 2]. Irreversible short-term and long-term adverse outcomes caused by PMI increase the clinical need for perioperative cardioprotection during major surgery.

Perioperative cardioprotection has been applied for many years in cardiac and non-cardiac surgery and consists of pharmacological treatments, including beta-blockers, statins, alpha-2 agonists, aspirin, inhalation anesthetics, noble gases, and opioids [3], and nonpharmacological treatments, such as ischemic preconditioning (IPC), remote ischemic preconditioning (RIPC), and remote ischemic postconditioning (RIPostC) [4]. However, perioperative cardioprotection in cardiac and non-cardiac surgery remains a debated topic. Recently, mesenchymal stem cell therapy, which depends on the ability of self-renewal and secretion of regenerative cytokines, has been incorporated into the main therapeutic approaches in the regenerative medicine of cardiovascular diseases [5]. However, the problem of storage and transportation, and the risks of inducing tumorigenesis and deformity need to be addressed [6]. Exosomes primarily contribute to the efficacy of stem cells and are stable, easily stored, and not rejected by the immune system [7]. Mesenchymal stem cell-derived exosomes (MSC-Exos) were developed as a kind of novel cell-free therapy. They preserve the main biological features and functions of the parent cells and exhibit a strong cardioprotective effect [8]. We reviewed the studies related to MSC-Exos to improve the treatment of myocardial ischemia and investigated their ability to provide perioperative cardioprotection.

## 2. Mechanisms Underlying Perioperative Myocardial Injury

PMI is a kind of myocardial ischemia that mainly occurs during or a few days after surgery and might occur due to the usage of intense analgesia. Nearly 80% of patients sustaining PMI only show symptoms based on cardiac troponin but lack other typical ischemic symptoms, such as chest pain and changes in the ECG [9, 10]. Few PMI patients present atherosclerotic plaque rupture with thrombus formation and distal embolization. The flow-mediated hypoperfusion and supply-demand imbalance of oxygen promote PMI [11, 12].

## 3. The Biological Characteristics of Mesenchymal Stem Cell-Derived Exosomes

**Fig. 1.**
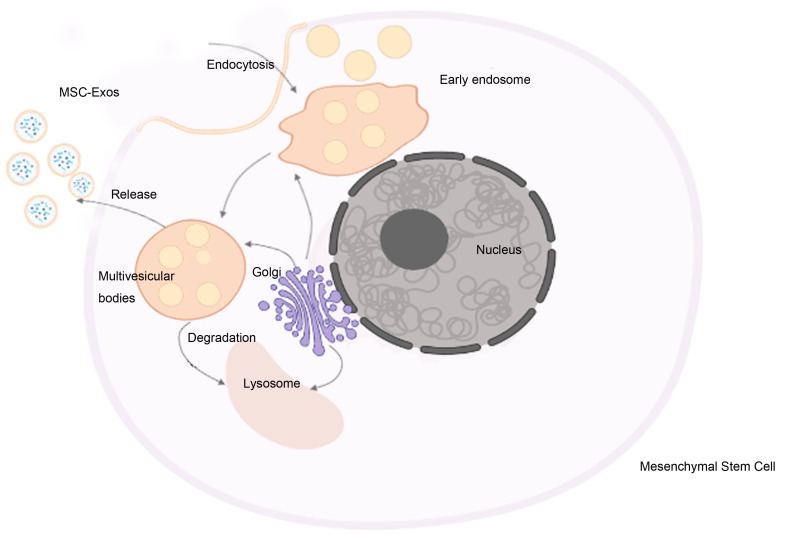
**The biogenesis of MSC-Exos**. First, the fusion of endocytic vesicles forms the early endosome. Then, early endosomes transform into multivesicular bodies. Finally, multivesicular bodies fuse with the plasma membrane to release exosomes via membrane budding. The MVBs might be transported to the Golgi for recycling endosomes and delivered to lysosomes for degradation.

Mesenchymal stem cells are found in many tissues, including adipose tissue, bone marrow, placenta, heart, peripheral blood, and umbilical cord [13]. They can regenerate by dividing and differentiating into several kinds of cells [14]. The application of MSCs in cardiovascular diseases has advanced considerably [5]. Exosomes, containing RNA, DNA, proteins, and lipids, are nano-sized lipid bilayer vesicles of endosomal compartments [15]. The biogenesis of exosomes is shown in Fig. 1. Besides having various exosome biogenesis-related functional proteins, MSC-derived exosomes contain surface markers, such as CD9, CD44, CD63, CD73, CD81, and CD90, specific markers of MSCs, proteins that act as signaling molecules [16, 17], and more than 850 unique gene products and miRNAs [18, 19]. Certain RNA cargos (mRNA and microRNA) that are sorted into MSC-derived exosomes are important for angiogenesis, cell differentiation, cell proliferation, cell survival, tissue remodeling, and immune system modulation [20, 21]. According to the results of RNA sequencing, MSC-Exos, isolated from different tissues, were found to have various species of tRNA [22] that affected the differences in the clinical efficacy of MSC-Exos. The five most abundant miRNAs in adipose-derived MSC (ASC) exosomes are miR-486–5p, miR-10a-5p, miR-10b-5p, miR-191–5p, and miR-222–3p. In bone marrow-derived MSCs (BMSCs), exosomes contain miR-143–3p, miR-10b-5p, miR-486–5p, miR-22–3p, and miR-21–5p. The miRNA sequencing data showed that the cardioprotection provided by endometrial MSCs was better than that provided by BMSCs and adipose-derived MSCs [23]; the cardioprotection-related miRNAs were upregulated (miR-29 and miR-24), while the cardiac-damage related miRNAs were downregulated (miR-21 and miR-15) [8, 24, 25].

## 4. Cardioprotection of Mesenchymal Stem Cell-Derived Exosomes

### 4.1 MSC-Exos Regulate Inflammatory Reactions 

**Fig. 2.**
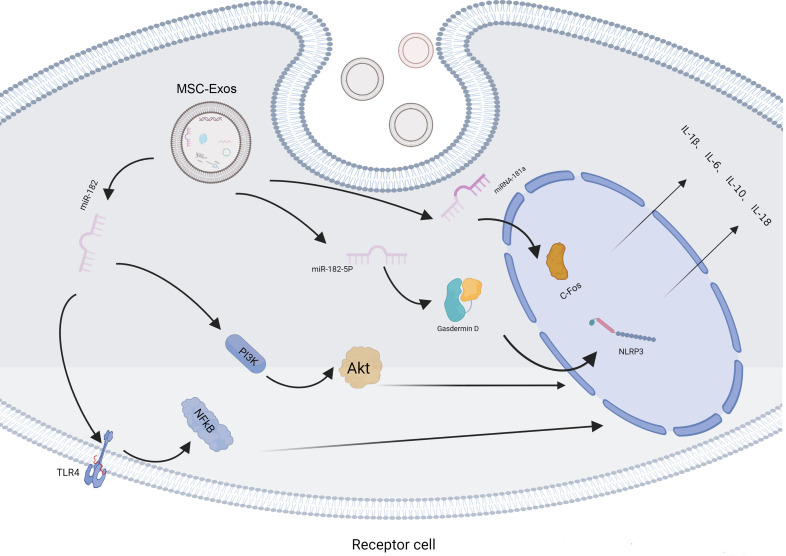
**MSC-Exos regulate inflammatory reactions in receptor cells**. The MSC-Exos enter the receptor cells and mediate the PI3K/AKT and TLR4/NF-kB signaling pathways, and the release of IL-1β, IL-6, IL-10, and IL-18 by transferring related microRNAs.

The inflammatory cascade plays a pivotal role in the myocardial ischemia-reperfusion (I/R) process [26]. The local inflammation induces pro-inflammatory cytokines and promotes cell proliferation and apoptosis [27, 28]. In turn, monocytes and macrophages secrete angiogenic cytokines and anti-inflammatory cytokines to promote injury repair [29]. MSC-Exo, the main efficient component of MSCs, participates in immune regulation [30]. Based on the myocardial I/R mouse model, Zhao and Fatih Arslan discovered that bone marrow-derived MSC-Exos could attenuate neutrophil infiltration [31, 32, 33], increase the concentration of the anti-inflammatory cytokine IL-10, and decrease the concentration of the pro-inflammatory cytokine IL-6 in the heart tissues of mice. More importantly, MSC-Exos promote the polarization of macrophages from the MI phenotype to the M2 phenotype by exchanging miR-182 to downregulate TLR4 and inhibit the relevant downstream signaling pathway (TLR4/NF-kB), while as the sequence of signaling cascade PI3K/Akt signaling pathway was activated, *in vivo* and *in vitro * [31]. MSC-Exos can increase the proportion of M2 macrophages by upregulating IL-10 and downregulating IL-6 via miR-21–5p, which reduces the inflammatory reaction in heart tissues [34]. MSC-Exos can deliver miR-182–5p and downregulate Gasdermin D to reduce the inflammatory cytokines (e.g., IL-1β and IL-18) released in the inflammasome of NLRP3 [35]. MSC-Exos enriched with miRNA-181a can attenuate inflammatory cell infiltration by targeting c-Fos, along with the upregulation of IL-10 and Treg cells and the downregulation of TNF-ɑ and IL-6 [36]. The basic mechanism is summarized in Fig. 2.

### 4.2 MSC-Exos Inhibit Cardiomyocyte Apoptosis and Autophagy

**Fig. 3.**
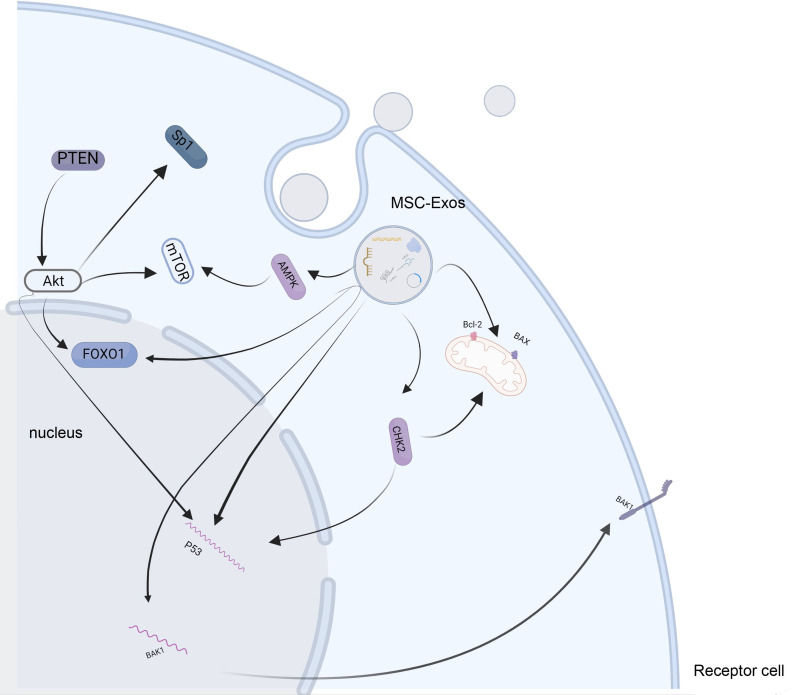
**MSC-Exos inhibit the apoptosis and autophagy of cardiomyocytes **. MSC-Exos enter the receptor cells that mediate the mTOR signaling pathway and increase the BCL-2/BAX ratio, thus regulating the expression of FOXO1 and p53.

Inappropriate apoptosis in ischemia strongly influences myocardial injury [37, 38, 39]. The phosphatidylinositol 3-kinase (PI3K)/protein kinase B (AKT) signaling pathway plays a pivotal role in myocardial cell apoptosis, which can be reversed by enhancer of zeste homolog 2 (EZH2) [40]. In hypoxia, bone marrow-derived MSC-Exos can ameliorate cardiomyocyte apoptosis [41]. Phosphatase and tensin homolog deleted on chromosome ten (PTEN), the target mRNA of miR-144, miR21, and miR-141, is downregulated in a hypoxic environment, which is reversed by bone marrow-derived MSC-Exos in a dose-dependent manner, and activates the downstream PTEN/p-AKT and PTEN/β-catenin signaling pathways [33, 42, 43]. In the mouse myocardial injury model induced by sepsis, a significant abundance of miR-141 was found in bone marrow-derived MSC-Exo-treated mouse myocardial tissues. Exosomal miR-141 targeted PTEN and activated β-catenin to alleviate myocardial injury. MiR-144 enriched in the bone marrow-derived MSC-Exo decreased PTEN expression, increased p-AKT expression, and prevented the apoptosis of H9C2 cells [42]. In turn, exosomes secreted from MSCs in a hypoxic environment enhanced the function of anti-apoptotic effects. MiR-125b increased the expression of the p53 and BAK1 mRNA [41]. Upregulating miR-221–3p and miR-146a-5p also inhibited the apoptosis of cardiomyocytes [44, 45]. MSC-Exos pretreated with macrophage migration inhibitory factor showed a strong cardioprotective effect. The transfer of lncRNA-NEAT1 between MSC-Exos and cardiomyocytes directly targeted miR-142–3p and regulated the expression of Forkhead Box O1 (FOXO1). Additionally, exosomal miR-183–5p could also target FOXO1, which can protect cardiomyocytes from apoptosis and cellular senescence effectively [46, 47, 48]. H9c2 cells treated with human umbilical cord MSC-Exo (hMSC-Exo) showed higher cell viability and inhibition of apoptosis and autophagy. High levels of Bcl-2 facilitate cardioprotection [49, 50, 51, 52]. In the studies conducted by Gu, *et al*. [50] and Zou, *et al*. [53] a high concentration of MSC-Exo enhanced the BCL-2/BAX ratio; thus, preventing the apoptosis of cardiomyocytes, increased the expression of Beclin-1, pAMPK, LC3II/I, and ATG13 and decreased the expression of P62 and Apaf1, activating the AMPK/mTOR-mediated autophagy flux pathway. However, according to a study by Li, *et al*. [54] exosomal miR-29c from bone marrow MSCs downregulated the LC3II/I ratio and the level of P62. Additionally, targeting PTEN activated the downstream AKT/mTOR signaling pathway, which prevented excessive autophagy in the myocardium. Activation of the CHK2-Beclin2 pathway regulated autophagy and attenuated the apoptosis of cardiomyocytes, which is targeted by exosomal miR-143–3p [55]. Additionally, the miR-143/Bcl-2/Beclin-1 axis is another pathway for decreasing cell apoptosis and inhibiting autophagy that is competitively bound by lncRNA UCA1 derived from hMSC-Exo [52]. In another Doxorubicin-induced myocardial injury model, miR-199a-3p enriched in MSC-Exo activated Akt; thus, inducing the expression of Sp1 and inhibiting the activation of p53, along with the overexpression of survivin to reduce apoptosis [51]. The main signaling pathways are shown in Fig. 3.

### 4.3 MSC-Exos Promote Angiogenesis

Myocardial injuries occur due to the dysfunction of angiogenesis and restriction of blood supply [56]. MSC-Exo has a robust proangiogenic ability, both *in vivo* and *in vitro * [57]. The GO analysis and the Panther pathway analysis aimed at the MSC-Exo proteome revealed canonical angiogenesis-related pathways, such as Fibroblast Growth Factor (FGF), Epidermal growth factor receptor (EGFR), Platelet-derived Growth Factor (PDGF), and cadherin [58]. In a study, Sun showed that MSC-Exo with abundant HIF-1a can increase the mRNA and protein levels of proangiogenic factors (e.g., VEGF and PDGF) and enhance neovessel formation to provide cardioprotection [59]. Hypoxic conditions can enhance this function [60]. Wang *et al*. [44] found that these proangiogenic effects were induced by the upregulation of miRNA-221–3p. *In vivo*, MSC-Exos were administered to ischemic limbs via intramuscular injection. Laser Doppler Perfusion Imaging showed that blood perfusion in limb ischemia was restored by nearly 85%, and the bioinformatics analysis suggested that proangiogenic effects might be induced by miR-7116–5p [61]. In the human umbilical vein endothelial cell model, MSC-Exo influenced capillary tube formation and promoted angiogenesis [60]. Although the mechanism is unclear, MSC-Exo can treat mouse hearts with a higher capillary density, which can protect the myocardium from ischemic injury [62, 63]. Intriguingly, Hemin (a potent heme oxygenase-1 inducer)-treated MSC-Exo had a superior effect in enhancing the capillary density compared to MSC-Exo [48]. Hemin pretreatment can upregulate miR-183–5p in MSC-Exo. Exosomal miR-183–5p can partially regulate the HMGB1/ERK pathway and inhibit ischemia-induced cardiomyocyte senescence to enhance the cardioprotective effects by regulating mitochondrial fission. Several experiments have confirmed that MSC-Exo can deliver miR-543 to reduce the expression of COL4A1 and lead to the proliferation, migration, invasion, and angiogenesis of cardiac microvascular endothelial cells [64].

### 4.4 MSC-Exo Participates in Cardiac Remodeling by Mediating Fibrosis

**Fig. 4.**
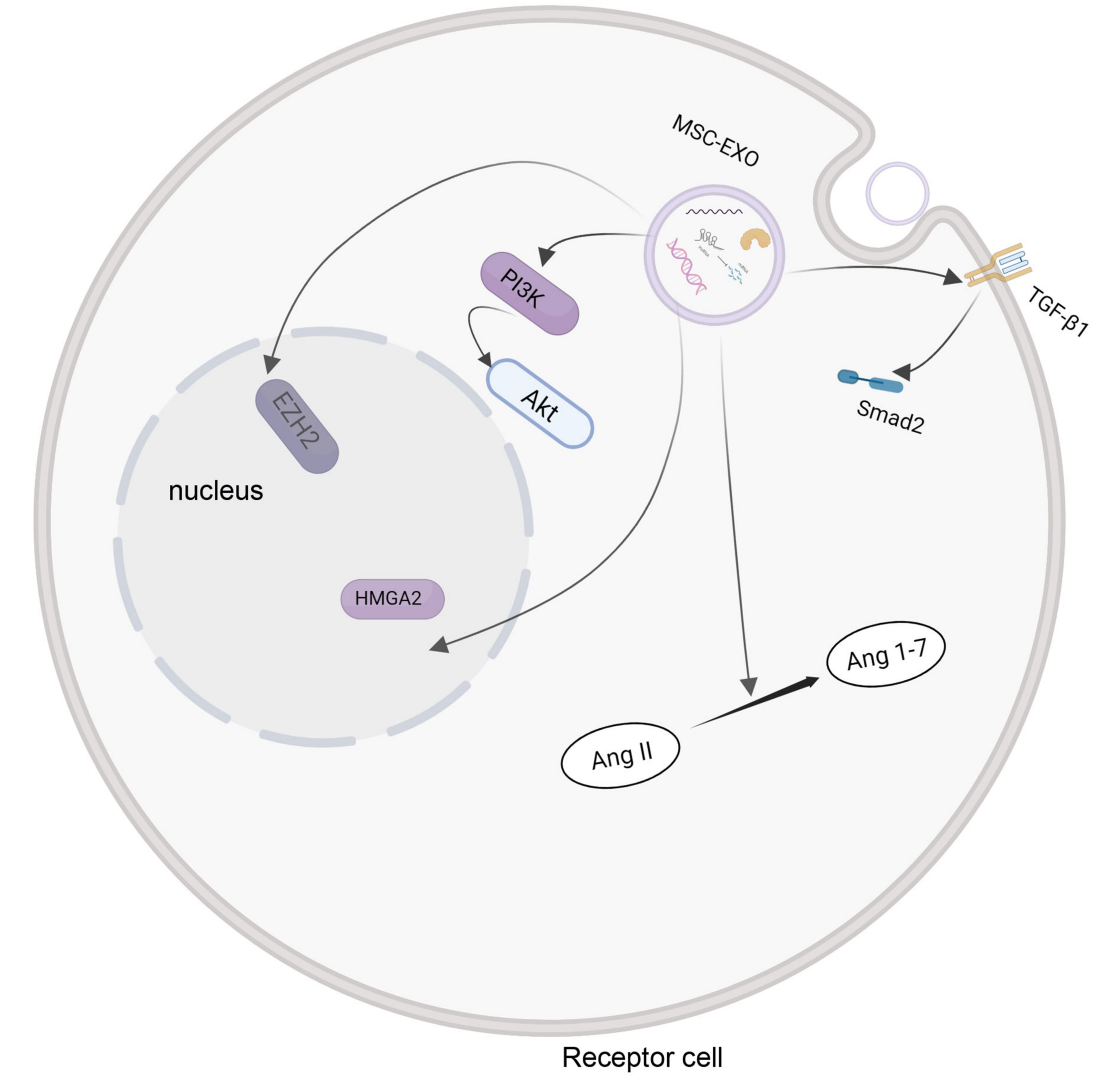
**The mechanism of cardiac remodeling is mediated by MSC-Exos **. MSC-Exos help to regulate the PI3K/Akt signaling pathway, the conversion of Ang II to Ang 1–7, and the expression of EZH2 and HMGA2.

Reactive fibrosis, followed by the loss of cardiomyocytes, occurs in most myocardial injuries and contributes to the remodeling of post-myocardial injury [65, 66]. Collagen I promoted myocardial fibrosis in myocardial injury [67]. MSC-Exo can alleviate myocardial fibrosis and improve cardiac function more effectively than MSC [8, 40, 68, 69]. In the epithelial-mesenchymal transition (EMT) process, epithelial cells are gradually transformed into mesenchymal cells. EMT facilitates the pathogenesis of fibrosis [70]. MSC-Exo can downregulate EZH2 and upregulate High Mobility Group AT-Hook 2 (HMGA2); thus, activating the PI3K/AKT pathway that can delay EMT and fibrosis in myocardial tissues, increase the left ventricular end-diastolic internal diameter (Dd), and end-systolic internal diameter (Sd), and increase the cardiac function [40]. In diabetic patients, MSC-Exo can reduce fibrosis and damage to the myocardial tissue by inhibiting the TGF-β1/Smad2 signaling pathway to decrease the expression of Smad2 and TGF-β1 proteins. Moreover, MSC-Exo can increase the level of fatty acid transporters and fatty acid beta oxidase [71]. Arslan, *et al*. [32] found that MSC-Exo can also preserve the structure and function of the left ventricle by activating the PI3K/Akt pathway, elevating the level of ATP and NADH, and attenuating oxidative stress. Additionally, the renin-angiotensin (RAS) system helps to improve the index of cardiac function and cardiac remodeling. MSC-Exo maintains the balance of the RAS system, promotes the translation from Ang II to Ang 1–7, and provides constant myocardial protection [72]. The mechanism of cardiac remodeling facilitated by MSC-Exos is shown in Fig. 4. The characteristics and molecular mechanisms of all the related studies mentioned above are shown in Table 1 (Ref. [31, 32, 33, 34, 35, 36, 40, 41, 42, 43, 44, 45, 46, 47, 54, 55, 61, 64, 71, 72]).

**Table 1.** **The characteristics and molecular mechanisms of the related studies**.

Study	Design	Myocardial injury model	Intervention	Result	Mediator	Signalling pathways
Zhao J 2019 [31]	mice	Ligating LCA	bone marrow-derived MSC-Exo	Converting macrophages to M2 phenotype and alleviating cardiac inflammation	miR-182	TLR4/NF-κB/PI3K/Akt
Arslan F 2013 [32]	mice	Ligating LCA	huES9.E1 derived MSC-Exo	reducing WBC count	activate adenosine receptors	PI3K/Akt
Pei Y 2021 [33]	mice	cecalligation puncture induced myocardial impairment	bone marrow-derived MSC-Exo	reducing the inflammatory infiltration and cell apoptosis	miR-141	PTEN/β-catenin
Shen D 2021 [34]	mice	Ligating LCA	C57BL/6 mouse derived MSC-Exo	promote the polarization of macrophages to the M2 phenotype	miR-21-5p	Not given
Yue R 2022 [35]	mice	Ligating LAD	bone marrow-derived MSC-Exo	Reducing GSDMD-dependent cell pyroptosis and inflammation	miR-182-5p	Not given
Wei Z 2019 [36]	mice	Ligating LAD	human umbilical cord blood-derived MSC-Exo	Reducing inflammatory cell infiltration	miRNA-181a	TNF-α and IL-6
Jiao W 2022 [40]	rat	Ligating LAD	bone marrow-derived MSC-Exo	Reducing fibrosis	EZH2	PI3K/AKT
Zhu LP 2018 [41]	mice	Ligating LAD	bone marrow-derived MSC-Exo	ameliorating cardiomyocyte apoptosis	miR-125b	p53 and BAK1
Wen Z 2020 [42]	H9C2 CMCs of rat cardiac origin	Cells were incubated in the hypoxic container for 48 h at ${\mathrm{37}}^{\circ }$C in a CO2 incubator	bone marrow-derived MSC-Exo	protect H9C2 cells from apoptosis	MiRNA144	PTEN/AKT
Shi B 2018 [43]	cardiac stem cells of the rat	CSCs are treated with 100 μM H2O2 for 2 h	bone marrow-derived MSC-Exo	protection against oxidative stress-triggered cell death	miR-21	PTEN/PI3K/Akt
Wang Q 2021 [44]	rat	Ligating LAD	human umbilical cord blood-derived MSC-Exo	Promoting the survival and angiogenesis in cardiomyocytes	miR-221-3p	Not given
Liu C 2021 [45]	mice	Cecal Ligation and Puncture	bone marrow-derived MSC-Exo	protect cardiomyocytes of inflammation model	miR-146a-5p	MYBL1
Chen H 2020 [46]	cells	Human-induced pluripotent stem cell (hiPSC)-derived cardiomyocytes	human adipose-derived MSC-Exo	protecting cardiomyocytes from apoptosis	miR-142-3p	LncRNA-NEAT1/miR-142-3p/FOXO1
Mao S 2022 [47]	rat	Ligating LAD	bone marrow-derived MSC-Exo	protecting cardiomyocytes from apoptosis	miR-183-5p	FOXO1
Li T 2020 [54]	mice	Ligating LAD	bone marrow-derived MSC-Exo	regulating autophagy under cardiac injury	miRNA-29c	PTEN/AKT/mTOR
Chen G 2021 [55]	rat	H9c2 cells were administrated to established the cellular hypoxia-reoxygenation model	bone marrow-derived MSC-Exo	Reducing cell apoptosis	miR-143-3p	CHK2-Beclin2
Ju C 2018 [61]	mice	Ligating LAD	cardiac derived MSC-Exo	Promoting cardiomyocyte proliferation, and preserves heart function	Not given	Not given
Yang M 2021 [64]	rat	Ligating LAD	Human mesenchymal stem cells derived exosome	Promoting cardiac microvascular endothelial cell angiogenesis	miR-543	COL4A1
Lin Y 2019 [71]	rat	diabetes mellitus-induced myocardial injury myocardial injury	bone marrow-derived MSC-Exo	Reducing myocardial injury and fibrosis	Not given	TGF-β1/Smad2
Xiao M 2021 [72]	rat	H9c2 cells	bone marrow-derived MSC-Exo	Improving cardiac remodeling and cardiac function	Not given	renin-angiotensin system

Note: left anterior descending coronary artery LCA; left anterior descending LAD.

## 5. Discussion

Exosomes are endocytic vesicles that play a key role in communication between cells. The biogenesis, uptake, composition, and physiological features have been discussed in previous reviews [73, 74, 75]. Although the exact mechanism is unknown, exosomes are extracellular nanovesicles mainly involved in cardioprotection. In a prospective clinical study executed in Policlinico Hospital of Bari and “G. Monasterio” Foundation of Massa showed that distinct exosomal proteins playing their roles of cardioprotection in older cardiac surgery patients regardless of surgery type [76]. Lucio Barile proved cardiac progenitor cells (CPC) derived exosome possessed the capacity to reduce cardiomyocyte apoptosis, enhance angiogenesis, and improve LV ejection fraction in the rat myocardial infarction model [77]. In-depth study revealed that pregnancy-associated plasma protein-A existed in CPC derived exosomes played a significant role in reducing scar size and improving ventricular function in rats’ permanent coronary occlusion model [78]. The data of Valentina Casieri’s research indicated ticagrelor can be leveraged to modulate release of anti-hypoxic exosomes from resident human cardiac-derived mesenchymal progenitor cells (hCPCs) [79]. It is remarkable that, recently, MSC-Exos were also shown to provide effective cardioprotection as a cell-free treatment [80]. In our review, we comprehensively analyzed the feasibility of the application of MSC-Exos in perioperative cardioprotection, as it can regulate inflammatory reaction [30], mediate cardiomyocyte apoptosis and autophagy [81], promote angiogenesis [57, 58, 60, 82], and improve cardiac remodeling [32].

Some clinical research organizations conducted a series of exosome-related clinical trials. In a study, Dai, *et al*. [83] reported that ascites-derived exosomes (Aex) were administered in the immunotherapy of colorectal cancer in phase I clinical trials. Aex combined with Granulocyte-macrophage Colony Stimulating Factor (GM-CSF) was shown to have strong antitumor effects. Subsequent clinical trials showed that Dendritic cell-derived exosomes (Dex) have strong antitumor effects in melanoma and non-small cell lung cancer [84, 85, 86]. From a clinical perspective, MSCs have beneficial curative effects in some non-neoplastic diseases. The phase II/III clinical pilot studies in Sahel Teaching Hospital showed that MSC-Exos applied to grade III-IV chronic kidney diseases can inhibit inflammatory immune reactions and improve kidney function [87]. Moreover, clinical trials on bronchopulmonary dysplasia, macular holes, type 1 diabetes, and acute ischemic stroke are underway. Due to large inter-individual variability and technological limitations, MSC-Exos have not been widely applied in clinical treatment. Fortunately, other applications of exosomes in oncologic therapy have verified the safety and effectiveness of MSC exosomal therapy.

The bioactive cargoes in MSC-Exos are also being investigated. Several studies have shown that exosomal miRNAs and proteins are responsible for the cardiovascular protection and repair of MSC-Exos [21]. Exosomal miRNA is an important bioactive cargo in MSC-Exo and is transferred to the recipient cells and specifically combined with the complementary mRNA target; thus, it can regulate the expression of related genes. The result of miRNA analysis based on the NanoString platform showed that the predictable top 23 miRNAs of human bone marrow-derived MSC-Exo targeting 5481 genes enriched in the PDGF, TGF-β, and Wnt signaling pathways were associated with angiogenesis and tissue remodeling [88, 89, 90]. Determining the exact mechanism of action and the specific target genes of these miRNAs is important for the clinical application of exosomes. Many well-constructed models have shown that modified exosomes can provide perioperative cardioprotection efficiently [82]. Because of unresolved confounding factors (e.g., complex exosomal component, complicated isolation process, elaborate exosome-loading mechanism, etc.), modification of the MSC-Exo based on bioengineering has not been performed. According to the identity of the specific bioactive cargo and research on the mechanism of biogenesis of exosomes, enhancing the function of MSC-Exo via genetic manipulation needs to be investigated in future studies for clinical application. Some studies have shown that lentiviral transfection and virus-free electroporation can be used to develop bioengineered exosomes [21, 91] with higher efficacy. Through this method, a low dose of exosomes can be used to achieve superior effects, thus compensating for the limitations of exosome isolation. To summarize, optimal ways for harvesting, modifying, and applying exosomes need to be investigated to reduce perioperative myocardial injuries in cardiac and non-cardiac surgeries.

## 6. Conclusions

MSC-Exos regulate inflammatory reactions, inhibit cardiomyocyte apoptosis and autophagy, promote angiogenesis, and mediate cardiac remodeling to prevent myocardial injury. MSC-Exos show therapeutic potential for ischemic cardiac injury and have a good application prospect in Cardioprotection. However, exosomes alone are not enough to reverse cardiac dysfunction after myocardial injury. Further study of the molecular mechanism can better guide the clinical transformation.

## Abbreviations

MSC-Exo, Mesenchymal Stem Cell-Derived Exosome; PMI, perioperative myocardial infarction; IPC, ischemic preconditioning; RIPC, remote ischemic preconditioning; RIPostC, remote ischemic postconditioning; I/R, ischemia-reperfusion; PI3K, phosphatidylinositol 3-kinase; Aex, ascites-derived exosomes; hMSC-Exo, human umbilical cord MSC-Exo; EMT, Epithelial to mesenchymal transition; Sd, end-systolic internal diameter; Dd, end-diastolic internal diameter; RAS, renin-angiotensin; BMSC, bone marrow-derived MSCs; ASC, adipose-derived MSCs; FOXO1, forkhead box O1; PTEN, phosphatase and tensin homolog deleted on chromosome ten; GM-CSF, granulocyte-macrophage colony stimulating factor; Dex, dendritic cell-derived exosomes; FGF, fibroblast growth factor; EGFR, Epidermal growth factor receptor; PDGF, platelet-derived growth factor; VEGF, vascular endothelial growth factor; HMGA2, high mobility group at-hook 2.
